# Classification of soybean frogeye leaf spot disease using leaf hyperspectral reflectance

**DOI:** 10.1371/journal.pone.0257008

**Published:** 2021-09-03

**Authors:** Shuang Liu, Haiye Yu, Yuanyuan Sui, Haigen Zhou, Junhe Zhang, Lijuan Kong, Jingmin Dang, Lei Zhang

**Affiliations:** College of Biological and Agricultural Engineering, Jilin University, Changchun, Jilin, China; Taipei Medical University, TAIWAN

## Abstract

In this study, the feasibility of classifying soybean frogeye leaf spot (FLS) is investigated. Leaf images and hyperspectral reflectance data of healthy and FLS diseased soybean leaves were acquired. First, image processing was used to classify FLS to create a reference for subsequent analysis of hyperspectral data. Then, dimensionality reduction methods of hyperspectral data were used to obtain the relevant information pertaining to FLS. Three single methods, namely spectral index (SI), principal component analysis (PCA), and competitive adaptive reweighted sampling (CARS), along with a PCA and SI combined method, were included. PCA was used to select the effective principal components (PCs), and evaluate SIs. Characteristic wavelengths (CWs) were selected using CARS. Finally, the full wavelengths, CWs, effective PCs, SIs, and significant SIs were divided into 14 datasets (DS1–DS14) and used as inputs to build the classification models. Models’ performances were evaluated based on the classification accuracy for both the overall and individual classes. Our results suggest that the FLS comprised of five classes based on the proportion of total leaf surface covered with FLS. In the PCA and SI combination model, 5 PCs and 20 SIs with higher weight coefficient of each PC were extracted. For hyperspectral data, 20 CWs and 26 effective PCs were also selected. Out of the 14 datasets, the model input variables provided by five datasets (DS2, DS3, DS4, DS10, and DS11) were more superior than those of full wavelengths (DS1) both in support vector machine (SVM) and least squares support vector machine (LS-SVM) classifiers. The models developed using these five datasets achieved overall accuracies ranging from 91.8% to 94.5% in SVM, and 94.5% to 97.3% in LS-SVM. In addition, they improved the classification accuracies by 0.9% to 3.6% (SVM) and 0.9% to 3.7% (LS-SVM).

## Introduction

Soybean, a legume crop, is an important source of proteins and fatty acids [1], the largest available source of feed proteins, and the second largest source of edible oils [2]. Soybean total production was 7.6 million tons in 2018 around world, and approximately 40% of the total production was in China [3]. Due to the large population and the long-time preference for soybean oil, China has the highest consumption of soybean, globally. Additionally, soybean is the primary protein source for pig feeds, which accelerates its consumption [4]. However, several diseases have seriously threatened the soybean yield and quality. For example, Frogeye leaf spot (FLS), caused by the fungus *Cercospora sojina* Hara (CSH), is a soybean foliar disease that causes yield losses and seed deterioration, and therefore economic losses. FLS epidemics can cause yield losses up to 60%. FLS is a polycyclic disease in which infection, symptom development, and reproduction may all be repeated multiple times throughout a single season [5]. Therefore, it is essential to detect and assess the extent of disease to estimate its economic impact and apply control strategies.

Conventionally, the detection of foliar diseases and their severity relies primarily on visual assessment or chemical methods. Agronomists manually checking the leaf color patterns, the size of the lesion area, and crown structures, such as crop density, the number, shape and distribution of leaves, the number of stems and branches of the crop, et al [6]. However, the visual assessment method is subjective and can be influenced by the empirical knowledge of observers [7]. As the types of crop diseases increase, the diseases that can be detected are limited. Besides, when multiple diseases have similar morphological symptoms, it becomes difficult for observers to accurately differentiate them. Conversely, chemical methods, including polymerase chain reaction (PCR) and enzyme-linked immune sorbent assay (ELISA), are highly sensitive and can accurately detect diseases. Despite they accuracy, chemical methods are time-consuming, labor-intensive, and destructive [8]. Moreover, hyperspectral imaging has been used to detect the severity of crop disease [7, 9–12], but requires extensive and time-consuming computations and training [13]. Hence, an appropriate, non-destructive, rapid, and high-efficient method remains warranted to evaluate the severity of crop diseases.

Developed for precision agriculture applications, the hyperspectral technique has attracted much attention for detecting crop diseases and estimating their severity [14, 15]. This method is objective, non-destructive, and can detect internal physiological changes of leaves under different stress conditions with high efficiency. It is also time-efficient, which helps to facilitate management strategies and improve productivity [16, 17]. Hyperspectral technique can be also used to conduct an in-depth examination of crop characters, such as crop cell structure, chlorophyll content, moisture content, trace element content, and light reflection and absorption characteristics. This may not be achieved using multispectral data, which have the relative coarse bandwidths [18]. Many studies have shown the feasibility and potential of hyperspectral reflectance data for evaluating the degree of crop diseases such as rice glume blight disease [19], black bean yellow mosaic disease [20], and citrus greening [21]. Recently, machine learning methods have been used to develop models for crop disease classification [22, 23], such as neural networks [24–26], support vector machine (SVM) [11], and least squares support vector machine (LS-SVM) [22, 27, 28]. SVM is a supervised machine learning algorithm used for classification and regression, whereas LS-SVM is an extension of SVM that usually transforms low-dimensional nonlinear data into high-dimension linear data to address the challenges associated with nonlinear data modeling [29]. To avoid overfitting, LS-SVM favors the structural risk minimization principle over the traditional empirical risk minimization principle used in conventional neural networks [30]. LS-SVM is faster and more accurate than SVM, and can be used in both linear and nonlinear multivariate analyses [31]. Hyperspectral data contain excessive, redundant, and highly correlated information across a large number of wavelengths, which can increase the number of calculations. Therefore, data dimensionality reduction, which optimizes and simplifies the entire wavelengths, is essential. Several methods have been used effectively to reduce the dimensionality of hyperspectral data, including algorithms of effective wavelength extraction such as competitive adaptive reweighted sampling (CARS) [32, 33], calculation of spectral indices (SIs) [34–36], and principal component analysis (PCA) [37, 38]. The CARS algorithm follows the Darwin’s evolution theory of the ‘survival of the fittest’ [39]. In this algorithm, the variable of each wavelength is considered as a single individual. During selection, only individuals with strong adaptability are retained. Meanwhile, during the wavelength selection process, multiple subsets of wavelength variables with large absolute value of regression coefficient are obtained by eliminating the wavelength with small regression coefficient. After obtaining multiple subsets of variables, the cross–validation method is then used to obtain the optimal subset of variables with the smallest root mean square error of the cross-validation (RMSECV), which is defined as the optimal wavelength subset [40, 41]. CARS is a practical approach used to select characteristic bands [42–44]. Spectral indices (SIs), obtained by combining reflectance values at two or more wavelengths or ranges of wavelength, are used to eliminate irrelevant information, thereby enhancing objects’ characteristics [45–47]. PCA is a multivariate statistical method that can efficiently reduce the dimensions of data while retaining useful information from the original data. All principal components (PCs) are independent of each other, which helps to eliminate the influence of redundant information in a high-dimension dataset. PCA can also reduce the high dependence on adjacent wavebands. Hence, several studies have reportedly employed PCA for data compression of hyperspectral data [18, 48, 49]. Reduction in redundancy and correlation improves the accuracy and reliability of the analysis results of hyperspectral data. Nevertheless, the aforementioned studies employed a single data dimensionality reduction method and ignored the importance of different feature extraction methods combination. To date, no studies have comprehensively classified different classes of crop diseases using both single and combined feature extraction methods. Other applications of different combination of feature extraction methods include spectral images classification [50, 51] and crop nutrient elements and physiological information detection, such as nitrogen status prediction of rice [52] and chlorophyll content estimation in rice [53] and wheat [54] plants. These studies illustrated that combined feature extraction methods is more superior than using only one method.

In this study, the single methods of SIs, CARS and PCA, and a combination of PCA and SI are used to extract the effective information on FLS. Furthermore, the performance of the effective wavelengths and SIs for FLS detection was compared. Hyperspectral technology has been used for crop diseases evaluation, however, the application of leaf hyperspectral reflectance in combination with various data dimensionality reduction methods to establish models for the classification of soybean FLS has not yet been reported. Consequently, the main objectives of this study are to (i) estimate FLS disease class using leaf hyperspectral reflectance analysis, (ii) evaluate the performance of feature extraction and modeling methods for detecting FLS disease class, and (iii) determine the feasibility of classifying FLS through machine learning methods using 14 datasets.

## Materials and methods

### Sample cultivation and inoculation

**Fig 1:**
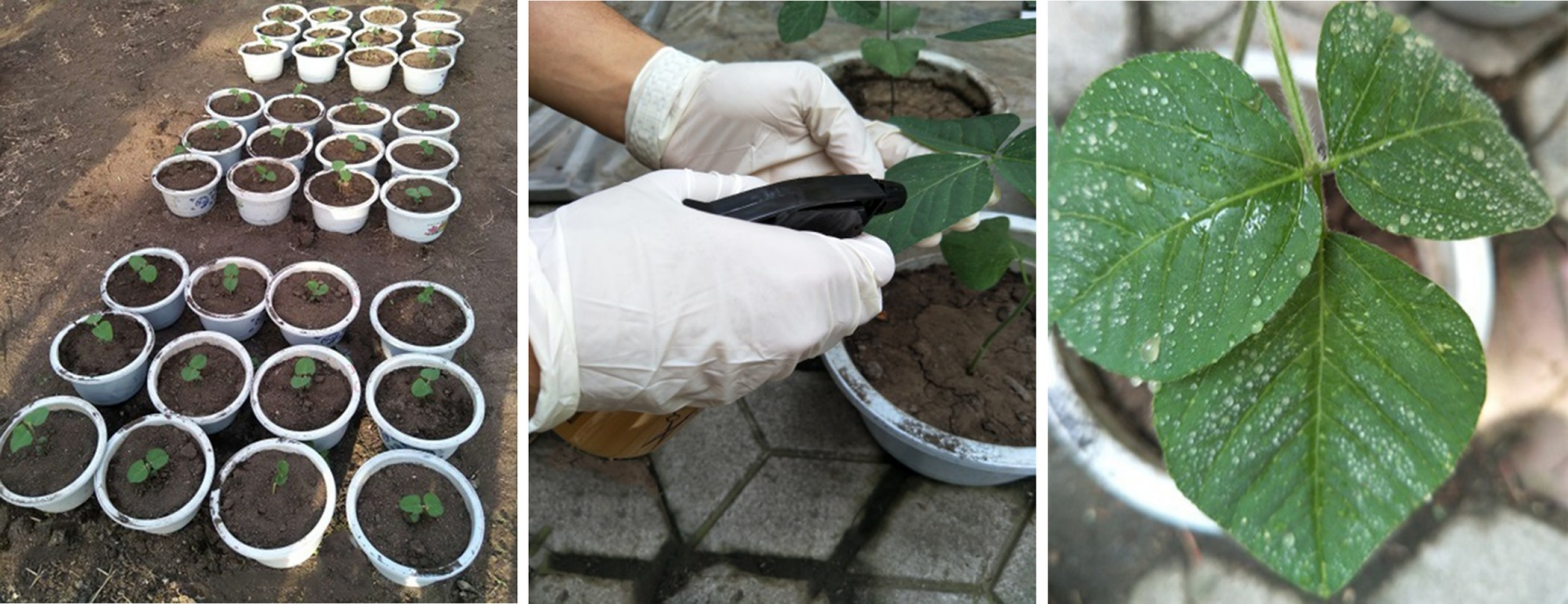
Representative images of soybean cultivation and inoculation. (a) Growing environment of soybean, (b) artificial inoculation, and (c) leaves post inoculation.

Soybean crops (Hushan 60) were grown in plastic pots (ø 260 mm) in an environmentally controlled solar greenhouse at the Jilin University, China, at 25/20°C (day/night), 60%-80% relative humidity (RH) and a photo-period of 15 h per day (Fig 1). Each pot held one plant, and a totally of 125 seeds were planted on 10 July 2019 (Fig 1A). Among them, 100 plants were utilized for infection, whereas the remaining 25 plants served as control. All plants were watered and fertilized normally in the same way before infection.

The inoculum of soybean FLS was obtained from the Jilin Academy of Agricultural Sciences, China. CSH conidia were harvested from potato dextrose agar cultures by flooding with sterile, distilled water and gently scraping the surface with sterile glass rods. The harvested conidia were, then, filtered with gauze, and 3% sucrose was added to produce an inoculation suspension with a spore concentration of 1×10^5^ ml^-1^. This inoculation suspension was used for infection. In total 100 plants were inoculated with the pathogens. Soybean crops were inoculated during their initial flowering stage on August 15, 2019. In this study, an artificial inoculation method was adopted, where whole plant leaves were sprayed uniformly with a small atomizer on cloudy days (Fig 1B). Fig 1C shows the state of leaves post infection. Following inoculation, the plants were covered with plastic bags for 48 h to maintain high humidity. The inoculation procedure was repeated after one week to ensure the incidence of FLS disease. Finally, the infected plants and control group were separated at different sites in two plastic sheds maintained at the same temperature at 28/18°C (day/night) and humidity (75%-80%).

### Data acquisition

The experimental data were collected between 10:00 and 14:00 on September 20, 2019. During the measurement was no wind, few clouds, and sufficient sunlight. In this study, two types of data, namely leaf images and leaf hyperspectral reflectance, were acquired. However, not all leaves infected with bacteria developed symptoms, which is common in FLS disease. The leaves with visible FLS were selected from the treatment group to obtain data, whereas healthy leaves were collected from the control group. One diseased or healthy leaf was regarded as one sample. In total, 440 samples, including 340 diseased and 100 healthy leaves, were collected and studied.

Before measuring the leaf hyperspectral data, one image each was taken for all the 440 samples. Leaf images were obtained using an ordinary mobile phone (OPPO R9s) with a resolution of 1600 million pixels. All photos were taken with a white background.

Leaf hyperspectral reflectance data were acquired using a Field Spec HandHeld-2 spectrometer (Analytical Spectral Devices, Boulder, Colorado, USA), which had a leaf clip accessory (from the same company) connected to it. The central part of each leaf was measured. The hyperspectral region ranged from 325 to 1075 nm, with a resolution of 3 nm. The number of hyperspectral channels is 512. The spectrometer was warmed up for 30 min before use to eliminate the influence of background on the spectral information, and the lamp was switched on for 5 min to maintain spectral stability. The spectrometer was calibrated to acquire the relative hyperspectral reflectance of the sample using the following equation:

$$R=\frac{R_{s}-R_{d}}{R_{w}-R_{d}}$$ (1)

Where R is relative reflectance, R_s_ is sample spectra, R_w_ is white reference, and R_d_ is dark current. The white reference was obtained with a circular white reference panel with a diameter of 3 inches, and the dark current was obtained by covering the lens with an opaque board. Ten reflectance curves per sample, and their average was calculated.

### Data processing

**Fig 2:**
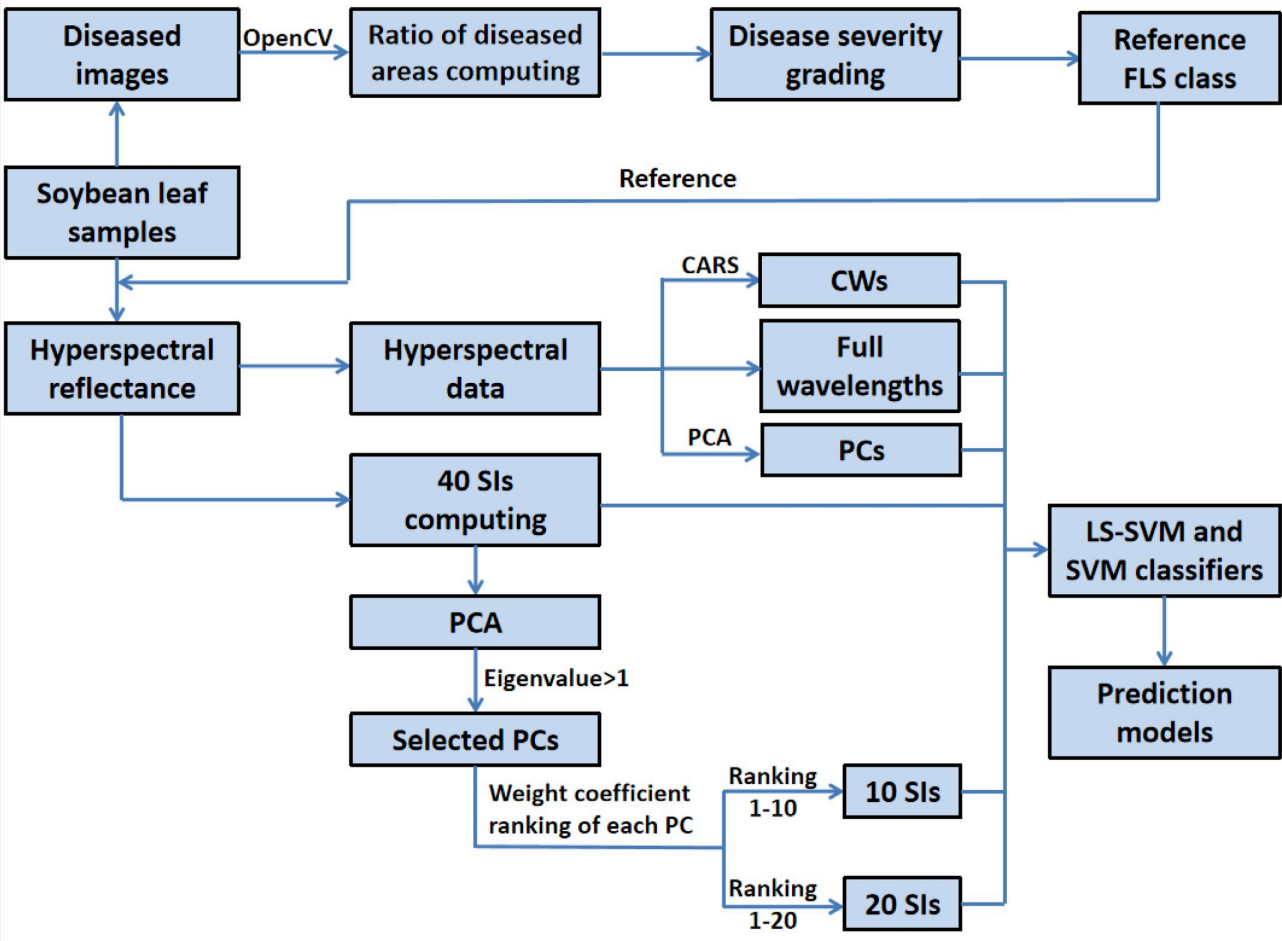
Flowchart of data processing and analyses methods used in this study.

The flowchart in Fig 2 presents of data processing methods performed in this study. The flowchart shows an image processing method and a hyperspectral reflectance data processing method to decrease hyperspectral dimensionality.

The severity class of the infection at the leaf scale depends on the lesion area on the leaf blade. The image processing method confirms the disease class by computing the ratio of the total leaf surface covered with the spot regions in the images of soybean leaves. This method was mainly based on the OpenCV function library, which uses computer vision recognition technology to extract the diseased area of FLS based on the hue, saturation, and value (HSV) color space of the image. Each diseased image was divided into two different areas based on the color feature. In HSV color space, pixels with threshold values greater than (1, 1, 1) and smaller than (37, 255, 255) were classified as FLS pixels, while those with values greater than (37, 255, 255) and smaller than (255, 255, 255) were classified as health pixels. Finally, the percentage of the total diseased area, with respect to the area of the whole leaf, was calculated. The values obtained were employed as a fundamental truth reference in this study, which will be referred to hereafter as the reference FLS class. The lesion ratio was divided into six classes based on the technical specifications for the evaluation of soybean frogeye leaf spot (Specification number: NY/T3114.2–2017) in China: 0%– 1% (class 1), 1%– 3% (class 2), 3%– 6% (class 3), 6%– 20% (class 4), 20%– 50% (class 5), > 50% (class 6). The healthy leaves were referred to as class 0.

In this study, hyperspectral reflectance data were processed using three single methods, namely, SI, CARS, PCA, and a PCA and SI combination method. These four methods were used to reduce the dimensionality of the hyperspectral data. First, we computed 40 SIs using two or more combinations of wavelength reflectance, and then conducted the selection of SIs by PCA based on the ranking of the weight coefficients. PCs and CWs were extracted from hyperspectral data on the full range of wavelengths using PCA and CARS respectively, at the same time. Finally, the selected SIs, PCs, and CWs were used to build classification models with the SVM and LS-SVM classifiers, and these models were compared with the model established by full range of wavelengths to assess the feasibility and superiority of the discrimination method of the FLS class employed in this study.

#### Spectral Index (SI)

This study listed 30 commonly used SIs from previous studies and 10 SIs developed in this study (Table 1). These SIs were derived from wavelengths in the visible and near-infrared regions and were calculated from the raw hyperspectral data after baseline correction, then, outlier were removed.

**Table 1:** List of spectral indices cited in previous literature and developed in this study.

SIs	Formulae	References
Normalized difference vegetation index	NDVI_1_ = (R900—R_680_)/(R_900_ + R_680_)	[55]
	NDVI_2_ = (R800—R_670_)/(R_800_ + R_670_)	[13]
	NDVI_3_ = (R870—R_530_)/(R_870_ + R_530_)	Present study
	NDVI_4_ = (R850—R_680_)/(R_850_ + R_680_)	Present study
	NDVI_5_ = (R855—R_650_)/(R_855_ + R_650_)	Present study
	NDVI_6_ = (R750—R_500_)/(R_750_ + R_500_)	Present study
	NDVI_7_ = (R815—R_715_)/(R_815_ + R_715_)	Present study
Modified triangular vegetation index	MTVI = 1.2*[1.2*(R800—R_550_) - 2.5 * (R670—R_550_)]	[13]
Renormalized difference vegetation index	$RDVI=\left(R_{800}-R_{670}\right)/\sqrt{R_{800}+R_{670}}$	[13]
Healthy-index	HI = [(R534—R_698_)/(R_534_ + R_698_)]- 0.5 * R_704_	[44]
Simple ratio	SR_1_ = R_800_/R_670_	[13]
	SR_2_ = R_800_/R_550_	[13]
	SR_3_ = R_750_/R_550_	[13]
	SR_4_ = R_850_/R_615_	Present study
	SR_5_ = R_800_/R_680_	Present study
	SR_6_ = R_780_/R_500_	Present study
Green index	GI = R_570_/R_670_	[56]
Disease-Water Stress Index	DSWI = R_550_/R_680_	[13]
Anth reflectance index	ARI = 1/R550−1/R_700_	[13]
Blue index	BI = R_450_/R_490_	[13]
Red-edge normalized difference vegetation index	Red-edge NDVI = (R750—R_705_)/(R_750_ + R_705_)	[57]
Optimized soil-adjusted vegetation index	OSAVI = (1 + 0.16) * (R800—R_670_)/(R_800_ + R_670_ + 0.16)	[58]
Pigment specific simple ratio chlorophyll b	PSSR_b_ = R_800_/R_635_	[13]
Pigment specific simple ratio carotenoids	PSSR_c_ = R_800_/R_550_	[13]
Redness index	RI = R_700_/R_670_	[13]
Normalized difference index	NDI_1_ = (R800—R_680_)/(R_800_ + R_680_)	[13]
	NDI_2_ = (R750—R_660_)/(R_750_ + R_660_)	[13]
	NDI_3_ = (R750—R_705_)/(R_750_ + R_705_)	[13]
	NDI_4_ = (R755—R_680_)/(R_755_ + R_680_)	Present study
	NDI_5_ = (R800—R_550_)/(R_800_ + R_550_)	Present study
Photochemical reflectance index	PRI_1_ = (R531—R_570_)/(R_531_ + R_570_)	[59]
	PRI_2_ = (R530—R_570_)/(R_530_ + R_570_)	[13]
Modified chlorophyll in reflectance index	MCARI = [(R700—R_670_) - 0.2*(R700—R_550_)]/(R_700_/R_670_)	[60]
Green normalized difference vegetation index	GNDVI = (R801—R_550_)/(R_801_ + R_550_)	[60]
Transformed chlorophyll absorption in reflectance index	TCARI = 3 *[(R700—R_670_) - 0.2*(R700—R_550_)/(R_700_/R_670_)]	[13]
Triangular vegetation index	TVI = 0.5 *[120 * (R750—R_550_)– 200 * (R670—R_550_)]	[13]
Blue green pigment index	BGI_1_ = R_400_/R_550_	[58]
	BGI_2_ = R_450_/R_550_	[58]
Blue red pigment index	BRI_1_ = R_400_/R_690_	[58]
	BRI_2_ = R_450_/R_690_	[58]

Note: R represents the hyperspectral reflectance, and the figure represents the corresponding wavelength.

#### Principal Component Analysis (PCA)

In this study, the PCA method was employed to extract effective PCs and select effective SIs.

The hyperspectral data were compressed by PCA, which extracted the effective PCs as input variables. The eigenvalue is an index indicating the magnitude of the influence of PC. In other words, the eigenvalue represents how much information of the original variables can be explained on average after PC is introduced. An eigenvalue of less than 1 for any given PC indicates that the effect of that PC is less important than that of a single variable. Therefore, only PCs with eigenvalues greater than 1 were selected for subsequent analysis in this study.

The abovementioned 40 SIs were subjected to PCA. In each selected PC, 40 SIs were arranged according to their weight coefficient. Each PC consisted of 40 SIs, where the weight coefficient of each individual SI represented its importance. Considering the principle, ‘sample number is 5–10 times greater than the optimal SI number’, the minimum and maximum numbers of SIs were regarded as lower and upper limits, whose components were scored in a column that was listed for each PC. Thus, the effects of the number of SIs can be evaluated in terms of their classification results.

#### Competitive Adaptive Reweighted Sampling (CARS)

CWs selection aims to select only a few wavelengths that carry the most useful information to decrease the computation of hyperspectral data [61]. In this study, all hyperspectral data were processed using the CARS algorithm; the selected wavelengths were CWs, which were treated as inputs to develop classification models.

### Modeling method

Two modeling methods, support vector machine (SVM) and least squares support vector machine (LS-SVM), were comparatively developed to identify and classify soybean FLS degrees in this study. The decision function of the SVM is:

$$f(x)=sgn(\sum_{i=1}^{n}y_{i}a_{i}K(x,x_{i})+b)$$ (2)

Where a_i_ is a Lagrangian multiplier, b is a deviation value, (*x_i_, y_i_*) is a support vector.

The decision function of the LS-SVM can be calculated as follows:

$$f(x)=\sum_{i=1}^{n}a_{i}K(x,x_{i})+b$$ (3)

Where a_i_ is a Lagrangian multiplier, K(x, x_i_) is a radial basis kernel function, and b is the statistic deviation.

In both SVM and LS-SVM models, a common factor, the kernel function, was expected to select. The radial basis function (RBF) kernel was recommended as the kernel function of SVM and LS-SVM, since RBF can handle the nonlinear relationships between the spectra and target attributes and provide a good performance under general smoothness assumptions. Thus, the RBF kernel was used as the kernel function of SVM and LS-SVM in this study. Two important parameters in the SVM model are the penalty coefficient (C) and the kernel function parameter gamma (g). C is the penalty coefficient, which is the tolerance for errors. g is a parameter that determines the nonlinearity of the RBF Kernel. This value implicitly determines the distribution of the data after it is mapped to the new feature space. In an LS-SVM model, there are also two important parameters to be determined: the regularization parameter gam (γ) and the parameter sig^2^ (σ^2^) of the RBF kernel function. γ determines the tradeoff between minimizing model complexity and minimizing the training error, and σ^2^ is the bandwidth, which implicitly defines the nonlinear mapping from the input space to a high dimensional feature space.

In this study, the k-fold cross-validation method was used to optimize the parameter combinations (C, g) and (γ, σ^2^). The training sample set was divided equally into k groups. Each time the (k-1) group was used for training, the other group was used for verification. Each group of data was rotated as verification data to verify the recognition rate of the model. K = 10 was used in this study. In the case of each set (C, g) and (γ, σ^2^), each group of data was rotated and verified. Generally, using more groups, increases the accuracies of the, calculations, but also increase the calculation time. Therefore, a trade-off between efficiency and accuracy is required. In addition, to achieve the optimal combination of (C, g) and (γ, σ^2^) and avoid overfitting challenges, a grid search method was employed.

In SVM and LS-SVM, dummy values, such as 0, 1, 2, 3, 4, were used as y values. The dummy values of 0, 1, 2, 3, and 4 were used to represent the five classes, i.e., FLS soybean classes of “class 0”, “class 1”, “class 2”, “class 3”, and “class 4”, respectively.

### Performance evaluation on models

The performances of both SVM and LS-SVM models were determined with the recognition rate of testing set as the classification accuracy of FLS classes for soybean. The classification accuracy gives an estimate of how well certain input variables performed. Two types of classification accuracy, the classification accuracy in each category, is called individual accuracy, and the other is overall accuracy, both of which are used to evaluate the models. The classification accuracy provided an estimate of how accurately a sample was classified, the higher the value, the better the model performance. The two accuracies were determined using Eqs (4) and (5) as follows:

$$individual\;accuracy\;(\%)=\frac{correctly\;classified\;samples\;in\;A}{all\;samples\;in\;A}\times 100$$ (4)

$$overall\;accuracy\;(\%)=\frac{the\;total\;number\;of\;correctly\;classified\;samples\;in\;each\;class}{all\;samples}\times 100$$ (5)

Where A represents the classes: ‘class 0’, ‘class 1’, ‘class 2’, ‘class 3’ and ‘class 4’, respectively.

### Data processing software

Data pre-processing, statistical calculations, and data analyses were carried out using the ViewSpec Pro (ASD Inc., Boulder, Colorado, USA), MATLAB R2018a (Mathworks Inc., Natick, USA), SPSS 24.0 (IBM Inc., Chicago, IL, USA) and Origin 19.0 (OriginLab, Hampton, USA). The hyperspectral curves were averaged using the ViewSpec Pro. FLS recognition and calculations were completed using MATLAB R2018a. SVM and LS-SVM models were set up using the libsvm toolbox and LS-SVM v1.8 toolbox running on MATLAB R2018a, respectively. SIs were selected through PCA using SPSS 24.0, and all graphs were drawn using Origin 19.0. All operations were conducted using Microsoft Windows 10 (64-bit) platforms.

## Results and discussion

### FLS areas extraction and classification

**Fig 3:**
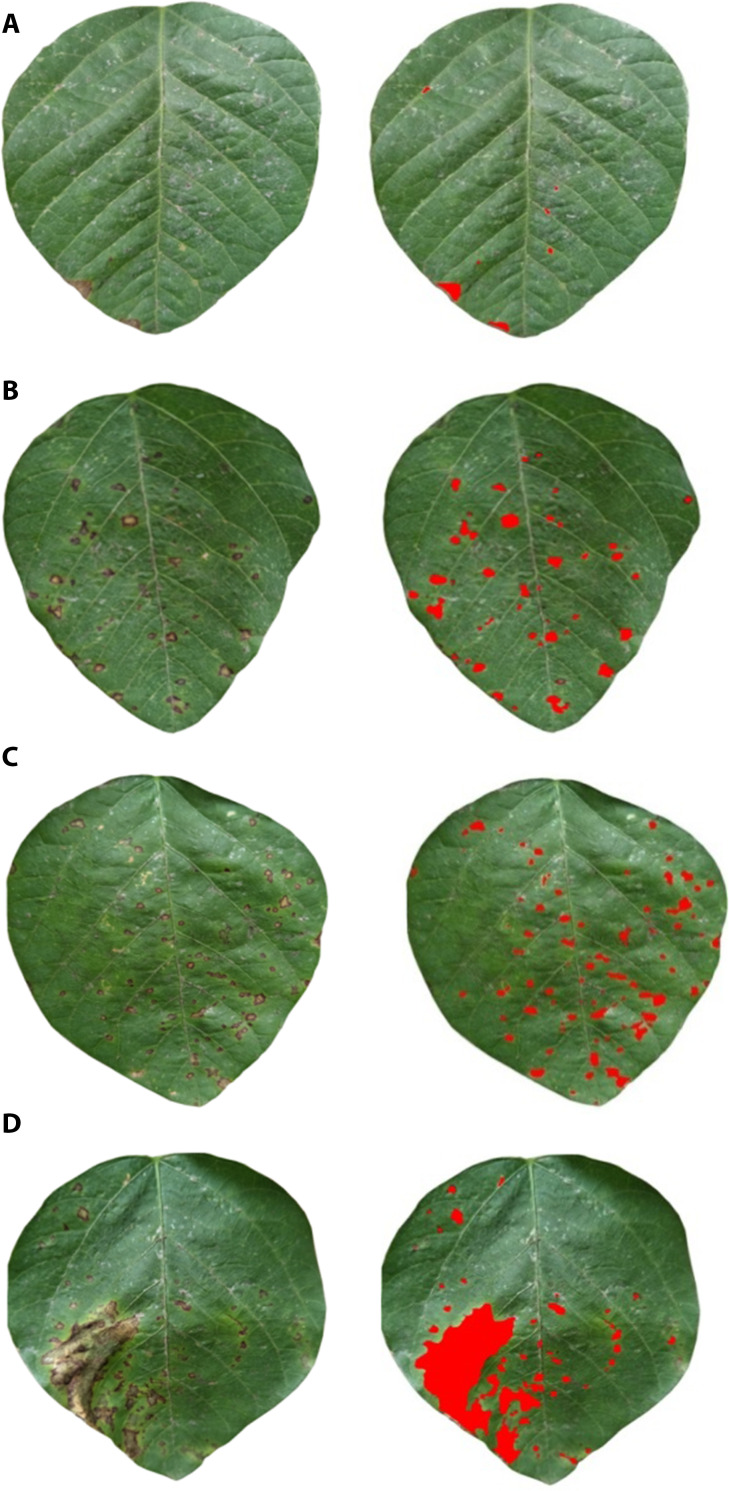
Original and processed images and, the proportion of FLS areas and classification results. (a) 0.43%, class 1, (b) 2.62%, class 2, (c) 3.41%, class 3, and (d) 10.87%, class 4. FLS areas were painted red on the leaves. Each class is represented by one figure.

Fig 3 shows the original and processed FLS images, the proportion of FLS areas and classification results based on computer machine vision technology. All 440 soybean leaves were divided into degrees 0–4 according to the proportion of FLS regions. The number of leaves in classes 0, 1, 2, 3, and 4 were 100, 100, 100, 80, and 60, respectively. Then, subsequent analysis of hyperspectral data was conducted based on the classification results as the reference FLS class.

### Signature of hyperspectral reflectance

**Fig 4:**
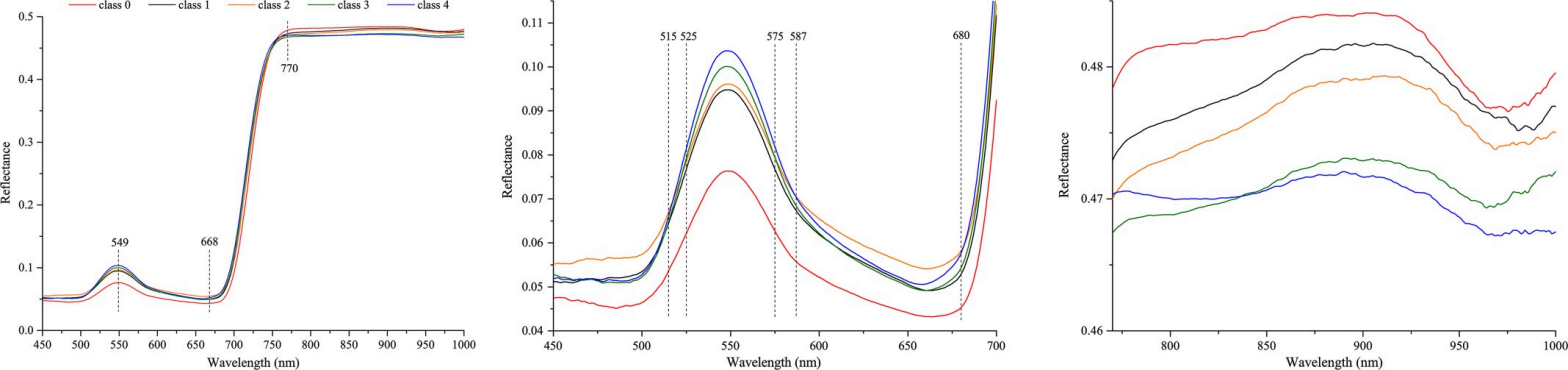
Hyperspectral curves of five classes of soybean FLS leaves. (a) Hyperspectral ranges of 450–1,000 nm, (b) zoomed in hyperspectral ranges of 450–700 nm, and (c) zoomed in hyperspectral ranges of 770–1,000 nm.

Fig 4 illustrates the hyperspectral curves of the five classes of soybean FLS leaves after a baseline correction was performed (Eq (1)). Only hyperspectral wavelengths in the range of 450–1,000 nm were displayed since regions in the beginning and end of the complete wavelength range had noisy signals. These five hyperspectral curves showed similar profiles and trends with a peak at approximately 549 nm and a valley at approximately 668 nm (Fig 4A). The reflectance increased sharply from 668 nm and reached the highest point at 770nm, and a relatively high reflectance was maintained up to 1,000 nm.

Reflectance in the visible (450–700 nm) and near-infrared (770–1,000 nm) bands were enlarged to observe the hyperspectral curves in detail (Fig 4B and 4C). As shown in Fig 4B, the hyperspectral of the FLS diseased leaves (class 1–4) were all considerably higher than that of the healthy leaves (class 0). The reflectance in the visible range was mainly affected by the chlorophyll content [46]. The leaves infected with FLS contained less chlorophyll and absorbed less green light and, thus, had higher reflectance in the visible range than healthy leaves. The four curves for the infected leaves (for class 1–4) intersected in the range of 512–525 nm, 575–587 nm, and 680–1,000 nm. Furthermore, the reflectance curves of classes 1, 3, and 4 intersected throughout the ranges of 450–525 nm and 575–700 nm. As shown in Fig 4C, the reflectance of healthy leaves (class 0) was higher than that of infected ones (class 1–4) in the near-infrared region. This was mainly due to damage or collapse of soybean leaf cell structure with the spread of FLS. Cells in diseased leaves were damaged and the flatness of the leaf surface was greatly reduced. Therefore, the incident light would have an irregular or diffused reflection from the leaf surface, which weakens the spectral signal received by spectrometer, thereby reducing reflectance in the near-infrared region. These results were similar to previous findings [62, 63]. However, it is challenging to visually differentiate and classify the classes of FLS in soybean leaves based on the hyperspectral reflectance covering the entire wavelength region of 450–1,000 nm.

### Selection of PCs using PCA

PCA was applied to the hyperspectral curves for data volume reduction and feature information extraction. A total of 26 effective PCs were extracted. Their eigenvalues and cumulative contribution rates are listed in Table 2. The cumulative contribution rates of the effective PCs (PC1–PC26) were greater than 94%, indicating that the information contained in the wavelengths can be interpreted by studying effective PCs.

**Table 2:** Eigenvalues and cumulative contribution rates of effective PCs.

PCs	Eigenvalue	Cumulative contribution rate	PCs	Eigenvalue	Cumulative contribution rate
PC1	318.79	42.45	PC14	1.53	92.81
PC2	228.32	72.85	PC15	1.46	93.00
PC3	60.23	80.87	PC16	1.41	93.19
PC4	30.29	84.90	PC17	1.39	93.38
PC5	24.00	88.10	PC18	1.35	93.56
PC6	11.34	89.61	PC19	1.28	93.73
PC7	8.26	90.71	PC20	1.23	93.89
PC8	5.35	91.42	PC21	1.20	94.05
PC9	2.12	91.70	PC22	1.17	94.20
PC10	1.83	91.95	PC23	1.15	94.36
PC11	1.75	92.18	PC24	1.13	94.51
PC12	1.63	92.40	PC25	1.03	94.64
PC13	1.56	92.61	PC26	1.00	94.78

### Selection of SIs by PCA

**Fig 5:**
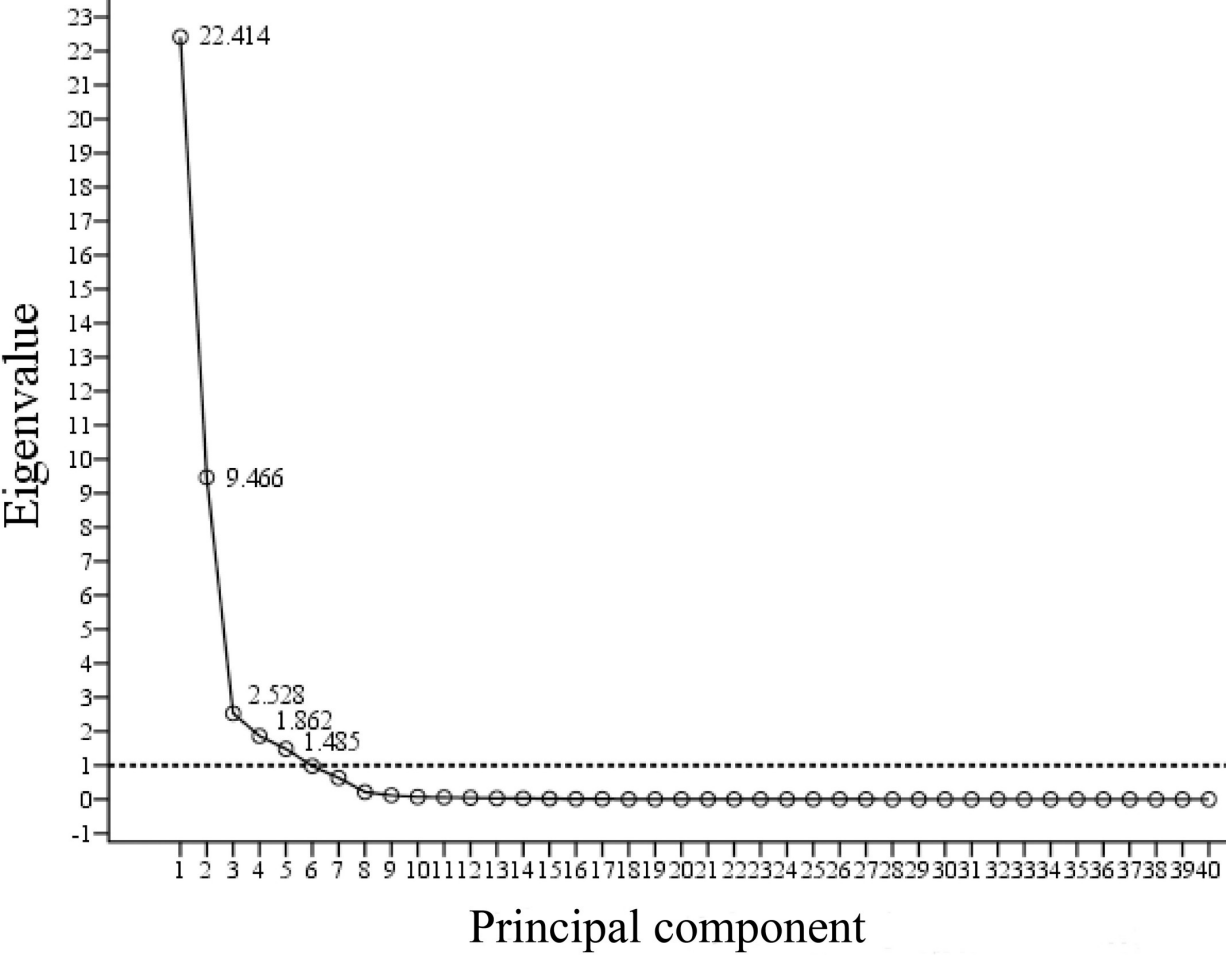
Principle components with their eigenvalues.

Since the maximum sample number in one class is 100, the optimal SI numbers of maximum and minimum are 20 and 10 respectively. PCs with eigenvalues greater than 1 and their component SIs ranking from 1 to 20 are shown in Fig 5 and Table 3.

**Table 3:** Five PCs and their component SIs ranking from 1 to 20.

PCs	SIs rank 1–10	SIs rank 11–20
PC1	PSSR_b_, SR_4_, NDVI_5_, NDI_4_, SR_5_, OSAVI, NDI_1_, SR_1_, NDVI_2_, NDI_2_	NDVI_4_, NDVI_1_, SR_3_, RDVI, NDI_5_, GNDVI, NDI_3_, Red-edge NDVI, SR_2_, PSSR_c_
PC2	RI, GI, DSWI, MCARI, TCARI, NDVI_6_, MTVI, SR_6_, TVI, NDI_2_	NDVI_2_, NDVI_1_, NDVI_4_, NDI_4_, NDI_1_, SR_1_, NDVI_5_, OSAVI, SR_5_, RDVI
PC3	ARI, BGI_1_, BRI_1_, SR_6_, NDVI_7_, NDVI_6_, PSSR_c_, SR_2_, GNDVI, SR_1_	NDI_5_, RI, SR_3_, NDVI_4_, SR_5_, SR_1_, NDI_1_, SR_4_, NDVI_2_, NDVI_5_
PC4	BRI_1_, BGI_1_, PRI_1_, PRI_2_, HI, DSWI, SR_6_, NDVI_6_, BRI_2_, GI	TVI, NDI_3_, Red-edge NDVI, SR_5_, NDI_4_, SR_4_, NDI_2_, BI, MTVI, SR_1_
PC5	BI, BRI_2_, MTVI, BGI_2_, TVI, RDVI, BRI_1_, BGI_1_, OSAVI, PSSR_b_	TCARI, GI, NDVI_1_, NDVI_4_, NDI_1_, DSWI, NDVI_3_, SR_5_, NDVI_7_, NDI_4_

The eigenvalues of the first five PCs were greater than 1, and were therefore used for following step (Fig 5). The first 20 SIs were listed for each PC, and were further divided into two groups: the first ten SIs and the second ten SIs according to their weight coefficient. Among the 100 SIs selected in all different PC combinations, the SIs with the highest frequencies were SR_5_, NDVI_1_, NDVI_4_, SR_1_, NDI_1_, and NDI_4_ (Table 3). These high-frequency SIs were associated with the wavelengths in the red and far-red region (670, 680, and 755 nm) and near-infrared region (800, 850, and 900 nm) (Table 1). The SIs with a frequency of only one were NDVI_3_, HI, ARI, PSSR_b_, PRI_1_, PRI_2,_ and BGI_2_ (Table 3). These low–frequency SIs had one or two wavelengths in the green region of 450–577 nm (NDVI_3_, HI, ARI, PRI_1_, PRI_2_, and BGI_2_) or at 635 nm (PSSR_b_) (Table 1). Only SIs ranking from 1 to 20 in each PC were selected as inputs in the classifiers for further data analysis since the other SIs were only slightly correlated with the reference classification.

### Selection of CWs by CARS

**Fig 6:**
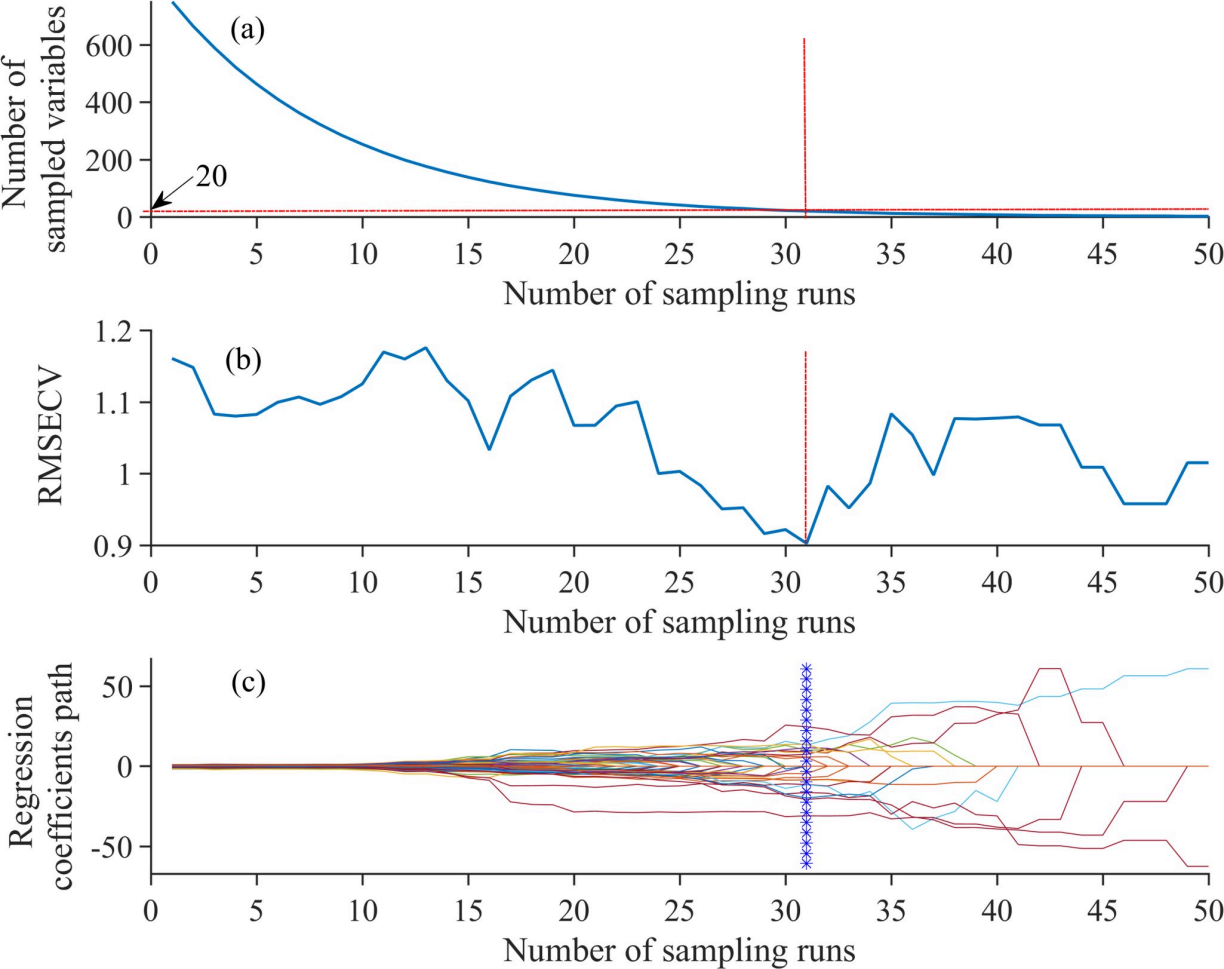
The altering trend of the CARS algorithm with the increasing of sampling runs. (a) The number of sampled variables, (b) the RMSECV values, and (c) the regression coefficients of each wavelength.

In CARS algorithm, there are two important parameters: the number of Monte Carlo sampling (MCS) and latent variables for cross-validation. The parameter of MCS number ranged from 10 to 100, while the parameter of latent variables ranged from 1 to 10 in this study. After several tests, the parameters of CARS were set as follows: the MCS number was fifty, the maximal number of latent variables for cross-validation six, and “center” was employed as the pretreatment method. After 50 runs, the value of the minimum root mean square error of cross-validation (RMSECV) was extracted (Fig 6). Fig 6A presents the relationship between the numbers of sampling runs and the reserved sampled variables. With an increase in sampling number runs, the number of selected wavelengths decreased and finally stabilized. As shown in Fig 6B and 6C, the RMSECV was first decreased as the irrelevant wavelengths were removed. At 31 sampling runs, the RMSECV attained a minimum value of 0.9026.

**Fig 7:**
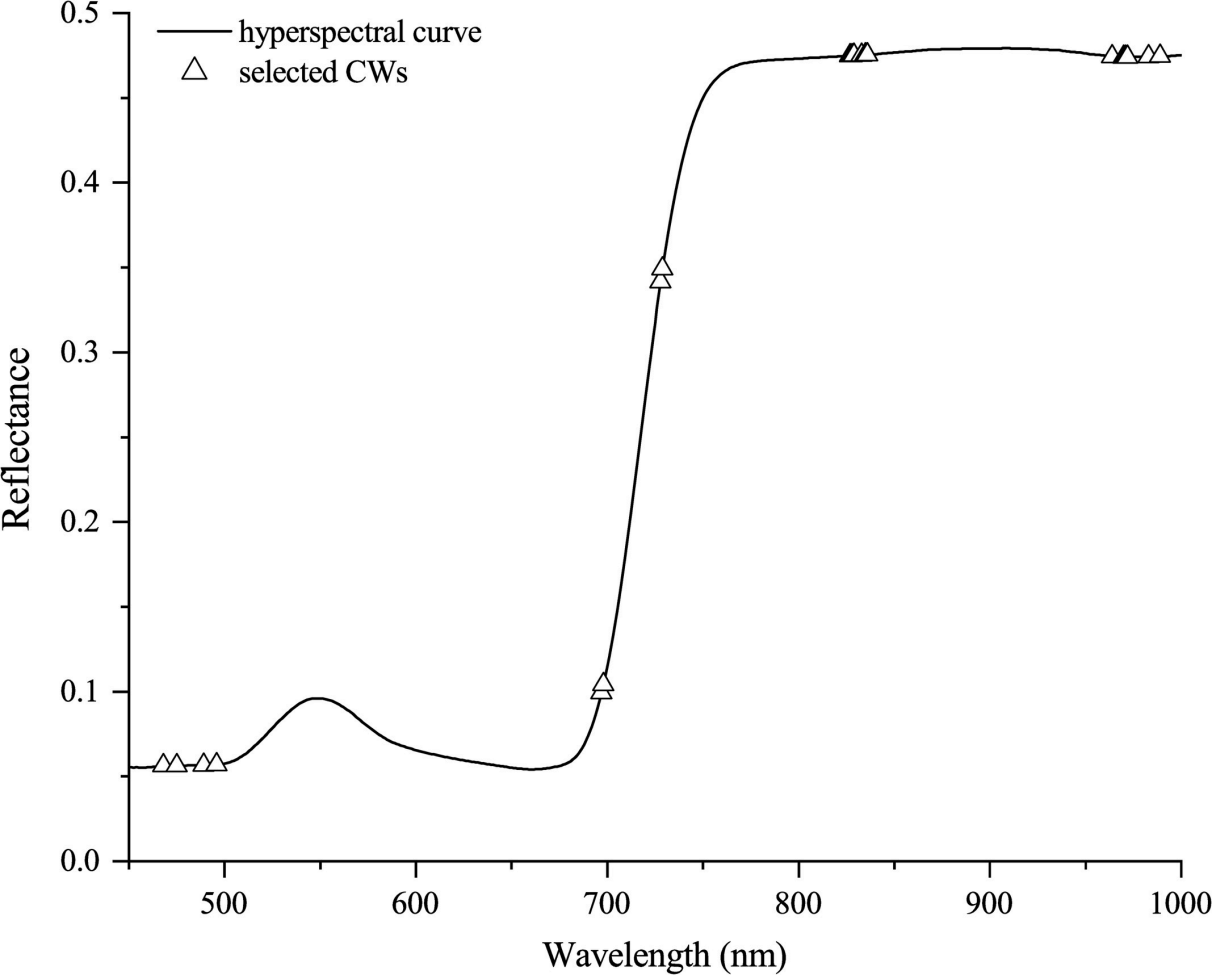
Distribution of characteristic wavelengths selected by CARS algorithm.

Twenty CWs (468, 475, 489, 496, 697, 698, 728, 729, 827, 828, 829, 833, 835, 836, 964, 970, 971, 972, 983, and 989 nm) were selected to determine the FLS class; their distribution is shown in Fig 7. The wavelength number decreased after selection of CWs using CARS, which considerably reduced the computation complexity.

Among the selected CWs, the wavelengths of 468, 475, 489, and 496 nm might have been associated with the absorption of anthocyanin in soybean tissue [64]. In addition, chlorophyll-b (620 nm) and chlorophyll–a (675 nm) showed characteristic peaks, which could explain the selection of 697, 698, 728, and 729 nm as CWs. Meanwhile, wavelengths at 827, 828, 829, 833, 835, and 836 nm may be linked to the absorption of nutrient elements such as nitrogen and zinc, while those at 964, 970, 971, 972, 983, and 989 nm, corresponding to the first and second O–H stretching overtones [65], were related to the water content. These findings suggest that CARS is useful for the selection of relevant wavelengths.

### Effects of data dimensionality reduction on classification accuracy

Table 4 presents the 14 datasets (DS1–DS14) placed into the SVM and LS-SVM classifiers directly to build classification models for FLS. Original full hyperspectral wavelengths are referred to as “Raw” in Table 4. Each of the five PCs contained two SI combinations (10SIs and 20SIs). Samples from each class were divided into a training set and a testing set with a ratio of 3:1. Hence, a total of 330 samples were selected as the training set, and the remaining 110 samples were used as the testing set (Table 5).

**Table 4:** The 14 datasets used to build SVM and LS-SVM classification models of FLS.

Raw	CWs	PCs	SIs	10SIs					20SIs				
				PC1	PC2	PC3	PC4	PC5	PC1	PC2	PC3	PC4	PC5
DS1	DS2	DS3	DS4	DS5	DS6	DS7	DS8	DS9	DS10	DS11	DS12	DS13	DS14

**Table 5:** Number of total samples, training set, and testing set in five classes.

Classes	Sample Number	Number of training set	Number of testing set
Class 0	100	75	25
Class 1	100	75	25
Class 2	100	75	25
Class 3	80	60	20
Class 4	60	45	15
Total	440	330	110

The classification accuracies of SVM and LS-SVM models for overall and individual class (class 0–4) with a total of 14 datasets, are illustrated and compared in Tables 6 and 7, respectively. The optimal parameters in the SVM and LS-SVM models were obtained using a grid-search procedure (Tables 6 and 7, respectively). Optimal parameters were used to achieve the optimal training model that predicted the testing samples. The ranges of γ and σ^2^ were set within 10^−2^–10^5^ in LS-SVM. The parameters C and g in SVM were set in the range of 2^−8^–2^8^. A total of 14 results were included in one class. The performances of different datasets varied from one another.

**Table 6:** Overall and individual class classification accuracies of SVM models with different datasets.

Dataset (DS)	Parameter (C, g)	Training set accuracy (%)						Testing set accuracy (%)					
		class0	class1	class2	class3	class4	overall	class0	class1	class2	class3	class4	overall
DS1	(16.21, 0.06)	100.0	100.0	100.0	100.0	100.0	100.0	88.0	100.0	92.0	70.0	86.7	90.9
DS2	(14.35, 3.28)	100.0	100.0	100.0	100.0	100.0	100.0	96.0	100.0	92.0	80.0	93.3	92.7
DS3	(4.05, 2.67)	100.0	100.0	100.0	100.0	100.0	100.0	100.0	100.0	92.0	75.0	86.7	91.8
DS4	(4.22, 8.73)	100.0	100.0	100.0	100.0	100.0	100.0	92.0	100.0	96.0	85.0	86.7	91.8
DS5	(8.21, 8.06)	100.0	100.0	100.0	100.0	98.3	99.7	76.0	88.0	88.0	70.0	86.7	81.8
DS6	(64.55, 4.43)	100.0	100.0	98.7	100.0	100.0	99.7	52.0	88.0	72.0	75.0	80.0	72.7
DS7	(16.14, 16.62)	100.0	100.0	98.7	98.3	97.8	99.1	72.0	72.0	68.0	70.0	66.7	72.7
DS8	(64.13, 16.02)	100.0	100.0	98.7	97.5	100.0	99.1	76.0	84.0	64.0	60.0	66.7	70.9
DS9	(4.38, 8.07)	100.0	100.0	100.0	100.0	100.0	100.0	64.0	88.0	80.0	70.0	73.3	76.4
DS10	(15.51, 2.33)	100.0	100.0	100.0	100.0	100.0	100.0	100.0	100.0	92.0	85.0	93.3	94.5
DS11	(4.65, 12.44)	100.0	100.0	100.0	100.0	100.0	100.0	96.0	100.0	96.0	85.0	86.7	93.6
DS12	(32.45, 16.06)	100.0	100.0	100.0	100.0	100.0	100.0	84.0	100.0	92.0	80.0	86.7	89.1
DS13	(8.54, 250.37)	100.0	100.0	100.0	100.0	100.0	100.0	76.0	100.0	92.0	80.0	80.0	86.4
DS14	(16.65, 8.77)	100.0	100.0	100.0	100.0	100.0	100.0	88.0	96.0	72.0	65.0	86.7	81.8

**Table 7:** Overall and individual class classification accuracies of LS-SVM models with different datasets.

Dataset (DS)	Parameter (γ, σ^2^)	Training set accuracy (%)						Testing set accuracy (%)					
		class0	class1	class2	class3	class4	overall	class0	class1	class2	class3	class4	overall
DS1	(7.54, 58.07)	100.0	100.0	100.0	100.0	100.0	100.0	100.0	100.0	92.0	85.0	86.7	93.6
DS2	(20.53, 7.63)	100.0	100.0	100.0	100.0	100.0	100.0	100.0	100.0	96.0	90.0	93.3	96.4
DS3	(26.46, 9.51)	100.0	100.0	100.0	100.0	100.0	100.0	100.0	100.0	92.0	85.0	93.3	94.5
DS4	(19.71, 3.07)	100.0	100.0	100.0	100.0	100.0	100.0	96.0	100.0	96.0	90.0	86.7	94.5
DS5	(7.26, 0.05)	100.0	100.0	100.0	100.0	100.0	100.0	84.0	100.0	84.0	75.0	73.3	84.5
DS6	(24.53, 4.22)	100.0	100.0	98.7	100.0	100.0	99.7	84.0	100.0	80.0	80.0	80.0	85.5
DS7	(13.58, 1.23)	100.0	100.0	100.0	100.0	100.0	100.0	80.0	88.0	76.0	80.0	73.3	80.0
DS8	(2.38, 4.82)	100.0	100.0	97.3	100.0	98.3	99.1	90.0	95.0	64.0	75.0	73.3	79.1
DS9	(8.98, 0.76)	100.0	97.3	98.7	100.0	100.0	99.1	92.0	76.0	76.0	75.0	80.0	80.0
DS10	(31.52, 2.11)	100.0	100.0	100.0	100.0	100.0	100.0	100.0	100.0	96.0	90.0	100.0	97.3
DS11	(36.38, 2.62)	100.0	100.0	100.0	100.0	100.0	100.0	100.0	100.0	96.0	95.0	93.3	97.3
DS12	(9.71, 11.06)	100.0	100.0	100.0	100.0	100.0	100.0	92.0	100.0	88.0	80.0	73.3	88.2
DS13	(11.20, 6.71)	100.0	100.0	100.0	100.0	100.0	100.0	96.0	92.0	88.0	70.0	86.7	87.3
DS14	(9.61, 7.58)	100.0	100.0	98.7	100.0	100.0	99.7	92.0	100.0	84.0	80.0	93.3	90.0

In the SVM models (Table 6), the overall classification accuracies of training sets and testing sets for different datasets varied between 99.1%–100% and 70.9%–94.5%, respectively. In general, most models performed well but, models built on DS10 are superior to others, with the classification accuracies of training and testing reaching 100% and 94.5%, respectively. The model constructed using DS8 obtained a relatively inferior performance with overall accuracy in both the training and testing sets (99.1% and 70.9%, respectively).

In the LS-SVM models (Table 7), classes 0, 1, 3, and 4 had the highest individual class accuracies (at 100%) of the testing set, whereas the highest accuracy for class 2 was 96%. Among all datasets, the lowest accuracies of classes 0–4 were 80%, 76%, 64%, 70%, and 73.3%, respectively. The difficulties in the classification of class 2 occurred in PC4 when 10 SIs were used (DS8), and the accuracy was 64%. In contrast, the classification accuracies for other classes were higher than 70%.

As shown in Table 7, the overall classification accuracies of the testing set for different datasets varied from79.1% to 97.3%. Most models gave satisfactory results when the overall classification accuracies were greater than 80%. The LS-SVM classification models established by DS8 and DS9 had the best prediction effect, with an accuracy of 97.3%. Compared with the model developed using the inputs of full wavelengths (DS1), which had an overall accuracy of 93.6%, five models showed better classification performance; these models were based on DS2, DS3, DS4, DS10, and DS11, and achieved overall accuracies of 96.4%, 94.5%, 94.5%, 97.3%, and 97.3%, respectively. They also improved the classification accuracy by 2.8%, 0.9%, 0.9%, 3.7%, and 3.7%, respectively.

Both SVM and LS-SVM classification models developed after feature extraction exhibited better performance with higher classification accuracies for both overall and individual class (Tables 6 and 7). All three single data volume reduction methods (SI, CARS, and PCA) improved the classification performance, which demonstrated the feasibility of using these methods in the present study. Further comparison was conducted with the combined feature extraction method that used PCA and SI. Ten combinations of PCs and SIs (DS5–DS14) were used as inputs. The two inputs based on DS10 and DS11 appeared higher accuracy for both overall and individual class. The cumulative 40 SIs of DS8 and DS9 were further analyzed, which showed that up to ten SIs (NDVI_1_, NDVI_2_, NDVI_4_, NDVI_5_, RDVI, SR_1_, SR_5_, OSAVI, NDI_2_, and NDI_4_) were coincident (Table 3). There were five high-frequency and no low-frequency SIs included among them. In addition, the wavelengths of these ten SIs were mostly in the red and near-infrared regions 660–855 nm, with, 670, 680, and 800 nm as the most common wavelengths (Table 1). These wavelengths are also related to plant vigor [66]. The results suggest not only red and near-infrared wavelengths contain more feature information, but are also responsible for the effectiveness of high-frequency SIs for the classification of soybean FLS in this study.

Both SVM and LS-SVM classification models with the worst overall performance were built using the same data set (DS8) and had the overall accuracies of only 70.9% and 79.1%, respectively. Eight SIs (BGI_1_, PRI_1_, PRI_2_, HI, DSWI, SR_6_, NDVI_6_, and GI) had at least one wavelength in the green region (500–570 nm) among the ten SIs used as inputs (Tables 1 and 3). However, these wavelengths were related to pigments, such as carotenoids and chlorophyll [36]. One study has shown no improvement in the models when using the blue and green spectral regions for input information [18]. In this study, we found that the leaf hyperspectral reflectance in the green region was poorly correlated with the soybean FLS classification. This was further confirmed as the wavelengths in the green region were not selected by CARS as CWs (Fig 7). This poor correlation could also be the reason the overall classification accuracy outcome was lowest for DS8.

The research findings of this study demonstrate that a combination of feature extraction methods (PCA and SI combinations) improve the classification performance for both SVM and LS-SVM models and enable the highest classification accuracies for both the overall and individual class. This combined method took advantages of both effective PCs and SIs rather than only one type of data dimensionality reduction method, such as only CWs, PCs, or SIs. Therefore, the combination of PCA and SI is an effective method for classifying soybean FLS, and a promising method for future studies.

### Comparison of SVM and LS-SVM classification models

**Fig 8:**
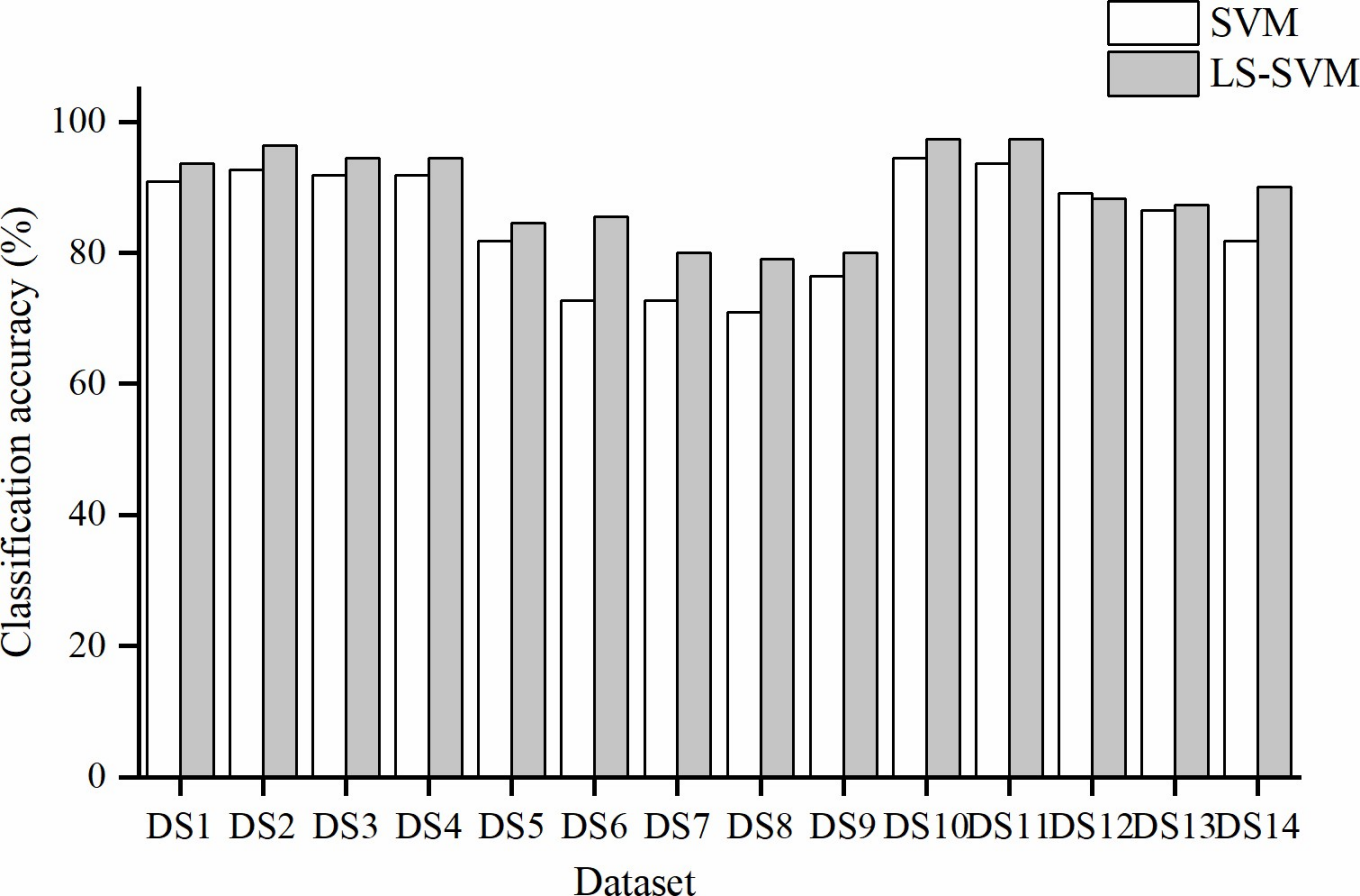
Performance comparison of SVM and LS-SVM classifiers.

The results of SVM and LS-SVM models along with the various feature extraction methods are illustrated in Fig 8. Comparing the FLS classification ability, we found that LS-SVM models generally presented higher classification accuracies than SVM models, and consistently outperformed SVM in soybean FLS class classification in this present study. LS-SVM better uses the latent nonlinear information of the hyperspectral data, which may have contributed to its better prediction performance.

## Conclusions

In summary, this study demonstrated the potential and feasibility of soybean FLS class estimation using hyperspectral technique. CARS, SI, PCA methods, and a combined method of PCA and SI were used to select 20 significant bands (CWs), 26 effective PCs, and more effective SIs to differentiate the various FLS classes. These methods extracted sensitive information related to FLS classes. Further, a combination of PCA and SI could extract more effective information from FLS than single methods. The LS-SVM classification models produced a more satisfactory performance after dimensionality reduction of hyperspectral data than the SVM models. The accuracies of the classification (for both overall and individual class) using DS2, DS3, DS4, DS10, and DS11 were greater than 90%, and using these datasets was more advantageous than using the complete hyperspectral data set (DS1). The combination models PC1-20SIs-LS-SVM and PC2-20SIs-LS-SVM, both with an overall classification accuracy of 97.3%, exhibited the best performances among all models built by SVM and LS-SVM. Furthermore, the data from the red and near-infrared regions was effective in differentiating the FLS disease classes. Our results provide a theoretical reference for improving disease monitoring systems.

## Supporting information

S1 Data Data of Fig 1. (XLSX)

S2 Data Data of Fig 5. (XLSX)

S3 Data Values of 40SIs in classes 0–4. (XLSX)

S4 Data Hyperspectral data used in this study. (XLSX)
